# ﻿Three new species of *Mitrephora* (Annonaceae) from Thailand

**DOI:** 10.3897/phytokeys.218.91582

**Published:** 2023-01-12

**Authors:** Charan Leeratiwong, Piya Chalermglin, Richard M. K. Saunders

**Affiliations:** 1 Division of Biological Science, Faculty of Science, Prince of Songkla University, Hat Yai, Songkhla, 90112, Thailand Prince of Songkla University Hat Yai Thailand; 2 Agricultural Technology Department, Thailand Institute of Scientific & Technological Research, 35 Technopolis, Liap Khlong Ha Road, Khlong Luang District, Pathum Thani Province 12120, Thailand Agricultural Technology Department, Thailand Institute of Scientific & Technological Research Bangkok Thailand; 3 Division of Ecology & Biodiversity, School of Biological Sciences, The University of Hong Kong, Pokfulam Road, Hong Kong,China The University of Hong Kong Hong Kong China

**Keywords:** Annonaceae, *Mitrephoralangsuanensis*, *Mitrephorasirindhorniae*, *Mitrephorasukhothaiensis*, new species, Thailand

## Abstract

Three new *Mitrephora* species (Annonaceae), *M.langsuanensis***sp. nov.**, *M.sirindhorniae***sp. nov.** and *M.sukhothaiensis***sp. nov.**, are described from Thailand. *Mitrephoralangsuanensis* resembles *M.macclurei*, but its leaves have more secondary veins, and its flowers have more carpels, with yellow-and-pink striped outer petals with a margin that becomes undulate with age. *Mitrephorasirindhorniae* resembles *M.tomentosa*, but has larger sepals and petals, longer flowering pedicels, and shorter monocarp stipes, with monocarps that have a longitudinal ridge. *Mitrephorasukhothaiensis* is distinct from its Thai congeners in having outer petals that reflex at maturity and inner petals with a pair of appendages on the margin adaxially. The addition of these three new taxa raises the total number of *Mitrephora* species in Thailand to 14. An identification key for Thai species is provided.

## ﻿Introduction

*Mitrephora* Hook.f. & Thomson (Annonaceae subfam. Malmeoideae tribe Miliuseae: Chatrou et al. 2012; Guo et al. 2017) is a horticulturally important genus of small tropical and subtropical trees that often bear attractive, flamboyant flowers. The only comprehensive taxonomic revision of the genus (Weerasooriya and Saunders 2010) recognised 47 species, although three additional species have since been described (Okada 2014; Damthongdee et al. 2019; Saunders and Chalermglin 2019).

The flowers are pendent and have two petal whorls, each of three parts. The inner whorl forms a mitriform dome (‘type III’ sensu Saunders 2010) that forms a floral chamber that is likely to function in secondary pollen presentation, capturing pollen that is released from the anthers and retained by hairs on the inner surface of the floral chamber (Saunders 2020). As with most Annonaceae species, the flowers are bisexual: although *Mitrephora* species are self-compatible, self-fertilisation is largely avoided by protogyny (Weerasooriya and Saunders 2010; Pang and Saunders 2014). *Mitrephora* species are likely to be pollinated by small beetles (Weerasooriya and Saunders 2010). The flowers are apocarpous and give rise to fruits comprising separate ‘monocarps’ that are derived from individual fertilised carpels.

Eleven *Mitrephora* species are currently recognised from Thailand (Weerasooriya and Saunders 2010; Damthongdee et al. 2019; Saunders and Chalermglin 2019; Weerasooriya et al. 2022), viz. *M.alba* Ridl., *M.chulabhorniana* Damth., Aongyong & Chaowasku, *M.keithii* Ridl., *M.macclurei* Weeras. & R.M.K.Saunders, *M.monocarpa* R.M.K.Saunders & Chalermglin, *M.sirikitiae* Weeras., Chalermglin & R.M.K.Saunders, *M.teysmannii* Scheff., *M.tomentosa* Hook.f. & Thomson, *M.vulpina* C.E.C.Fisch., *M.wangii* Hu, and *M.winitii* Craib. We describe three new species here, viz. *M.langsuanensis* Leerat., Chalermglin & R.M.K.Saunders, *M.sirindhorniae* Chalermglin, Leerat. & R.M.K.Saunders and *M.sukhothaiensis* Leerat., Chalermglin & R.M.K.Saunders.

The species descriptions provided here are based on observations from living plants (cultivated) and herbarium material (from BKF, HKU, KKU, PSU, QBG and SING herbaria). Taxonomic conclusions were based on comparisons with the extensive dataset generated by Weerasooriya and Saunders (2010). Data obtained from living plants (such as overall height) were obtained during the third growing season from four cultivated individuals of each species, grafted from top shoots. They were grown in full sunlight and irrigated.

## ﻿New species descriptions

### ﻿ Mitrephora langsuanensis

Taxon classificationPlantaeMagnolialesAnnonaceae

Leerat., Chalermglin & R.M.K.Saunders sp. nov.

2DBEF41F-E3E1-5CCC-914C-F9AC10109E2E

urn:lsid:ipni.org:names:77311807-1

Figs 1 , 2

#### Diagnosis.

*Mitrephoralangsuanensis* is similar to *M.macclurei* Weerasooriya & R.M.K.Saunders, but differs in having: leaves with more secondary veins (7–14 pairs), sometimes with domatia abaxially; yellow outer petals with pink stripes, densely hairy abaxially, and with a margin that becomes undulate with age; more carpels per flower (10–12); and longer fruiting pedicels (20–25 mm).

**Figure 1.**
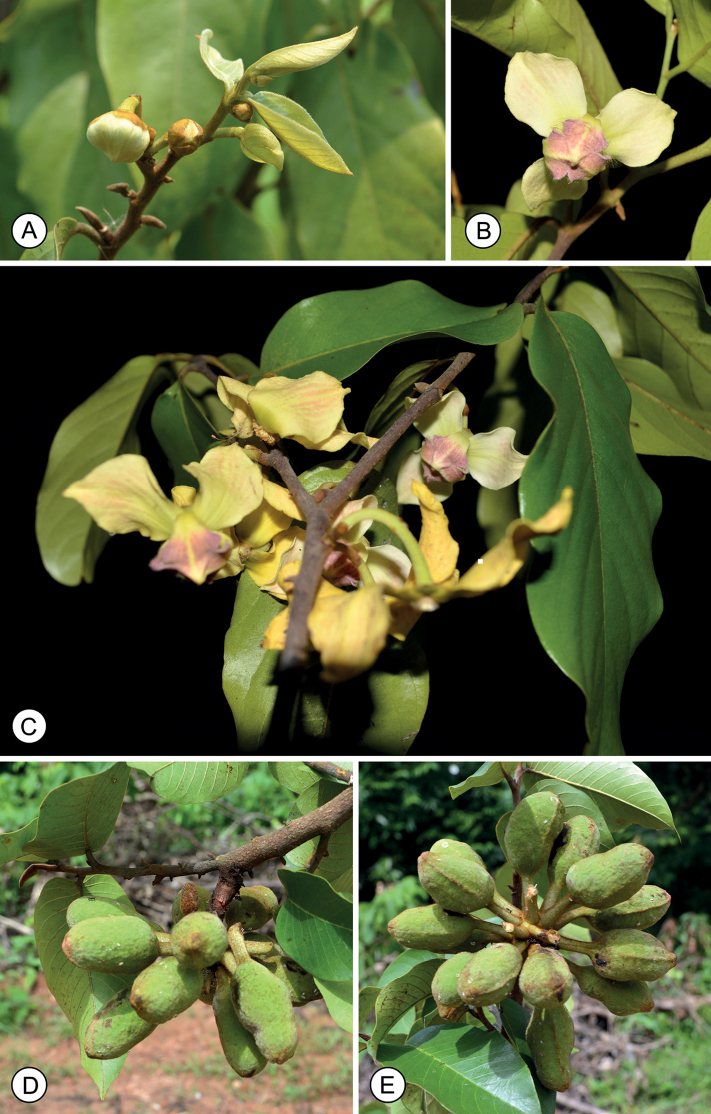
*Mitrephoralangsuanensis* sp. nov. **A** flower buds **B** flower **C** flowering branch **D, E** fruits (slightly immature). Photos by P. Chalermglin.

#### Types.

**Thailand**: TISTR Annonaceae collection plot, Khlong Luang District, Pathum Thani Province, Central Thailand, ca. 5 m alt., 3 May 2021, *P. Chalermglin 640503* (originally from Phu Muang temple, Lang Suan District, Chumphon Province, Peninsular Thailand, ca. 100 m alt.) (holotype PSU; isotypes BKF, KKU, QBG).

**Figure 2.**
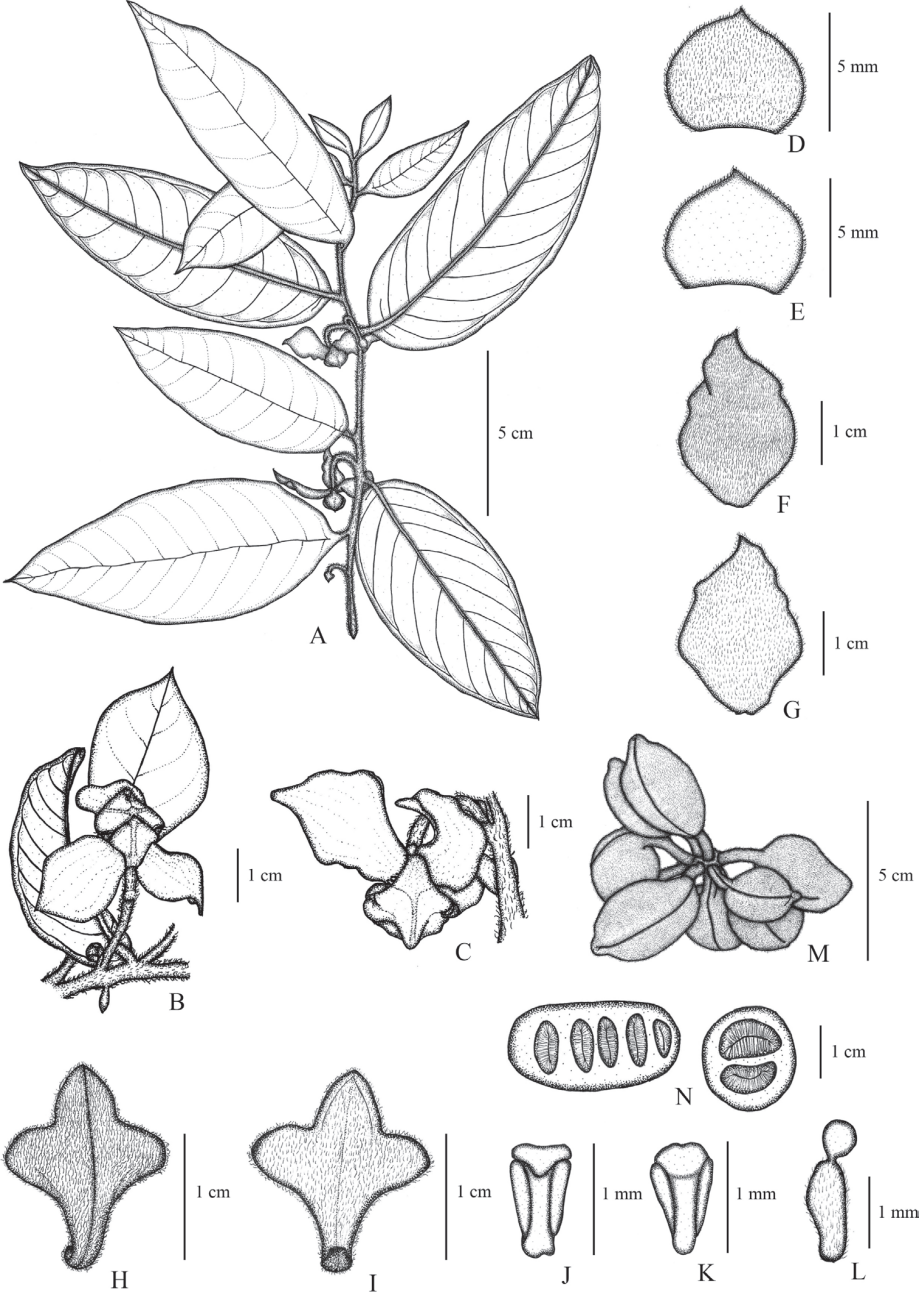
*Mitrephoralangsuanensis* sp. nov. **A, B** flowering branches **C** flower **D, E** Sepals (ab- and adaxial) **F**, **G** outer petals (ab- and adaxial) **H, I** inner petals (ab- and adaxial) **J, K** stamens (ab- and adaxial) **L** carpel **M** fruit, composed of separate monocarps **N** seeds (longitudinal and transverse sections). Drawn by A. Somphrom, from *P. Chalermglin 640503* (PSU).

#### Description

**(from cultivated material).** Small trees, to 4 m (in cultivation). Young branches densely pubescent. Leaf laminas coriaceous, (elliptic-)lanceolate, (5.5–)8–22 by 3–5 cm, base obtuse or rounded, apex acute to acuminate, glossy, glabrous adaxially, sparsely to moderately pubescent (sometimes pilose) abaxially, secondary veins 7–14 pairs per leaf, sometimes with domatia at axils of secondary veins abaxially; petioles 6–12 mm long, moderately pubescent. Inflorescence rachides simple, with rachis internodes remaining short, 2–4 mm long, 2–3-flowered, densely pubescent; pedicels 23–30 mm long. Sepals free, broadly ovate, not imbricate, 4–6 by 5.5–6.5 mm, densely pubescent abaxially, glabrous to sparsely pubescent, denser at margin adaxially. Outer petals creamy white, turning yellow with pink stripes, ovate, 20–27 by 15–17 mm, not clawed, margin undulate with age, acute, densely pubescent ab- and adaxially. Inner petals with greenish-yellow claw, purple towards apex with a yellow stripe, 13–15 by 9–11 mm, claw incurved, densely pubescent ab- and adaxially. Stamens 1.2–1.3 mm long, connective truncate, glabrous. Carpels 10–12 per flower, 2–2.7 mm long; ovary oblong, 1.3–1.6 mm long, moderately pubescent; stigma subglobose, 0.4–0.6 mm long, hairy; ovules 4–8 per carpel. Fruits with up to 12 monocarps, borne on a pedicel 20–25 mm long, 3–5 mm wide, densely pubescent. Monocarps yellow when ripe, ellipsoid, ovoid to oblong-ellipsoid, 20–40 by 17–20 mm, smooth, with longitudinal ridge, sparsely pubescent; stipes 16–20 mm long, moderately pubescent. Seeds 2–6 per monocarp, semi-lenticular (lowermost and uppermost within monocarp) or discoid (others), 10–13 by 5–8 mm, glabrous, pitted.

#### Phenology

**(in cultivation).** Flowering between May and June; fruiting in May.

#### Distribution and habitat.

**Figure 3.**
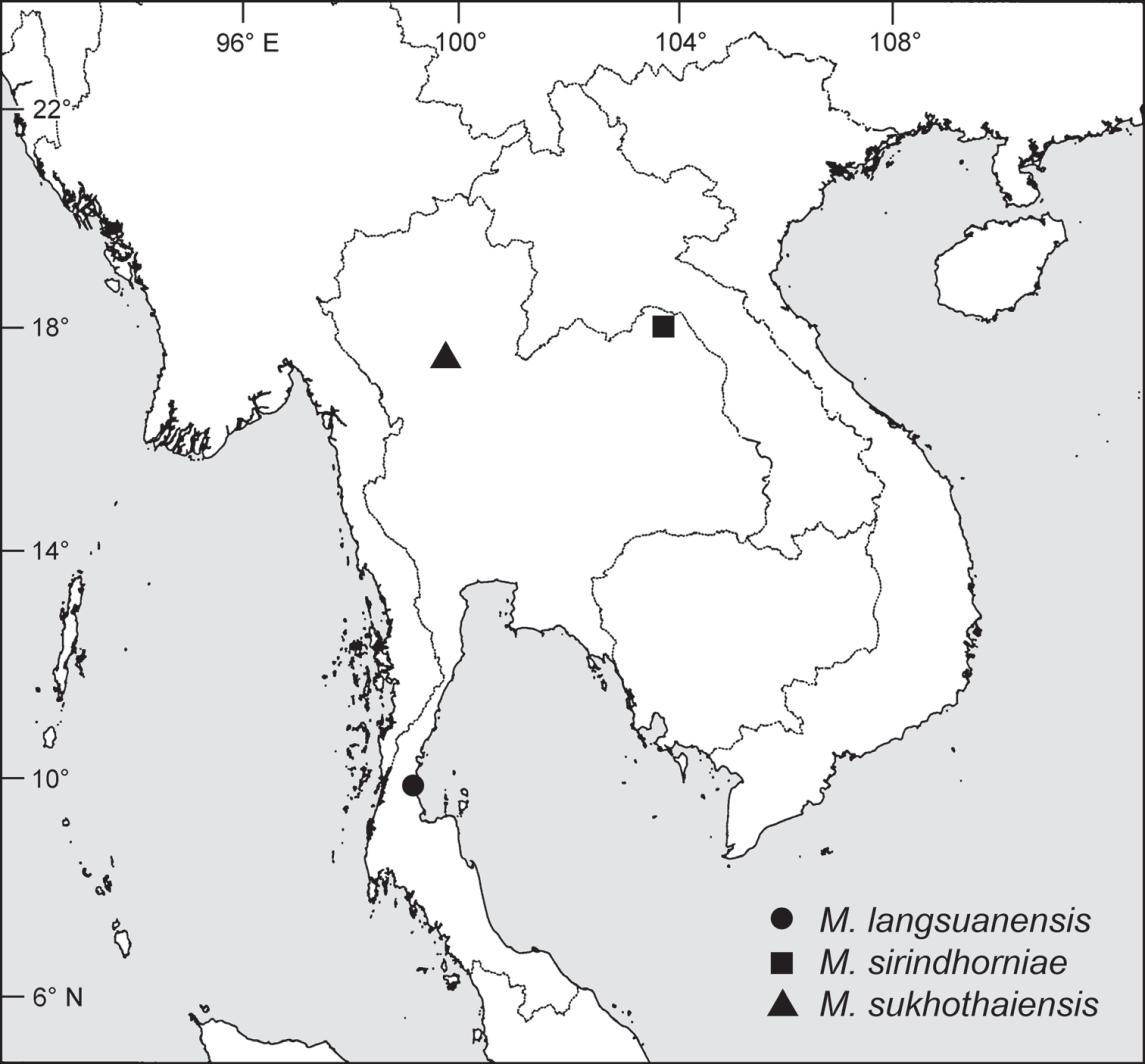
Distributions of *Mitrephoralangsuanensis*, *M.sirindhorniae* and *M.sukhothaiensis*.

Endemic to Chumphon Province, Peninsular Thailand (Fig. 3). Growing on limestone hill in tropical rain forest; ca. 100 m alt.

#### Etymology.

From the name Lang Suan district, Chumphon Province.

#### Local name.

Phrom lang suan (พรหมหลังสวน) (Chumphon).

#### Additional specimens examined

**(paratypes). Thailand**: Pathum Thani Province, Khlong Luang District, TISTR Annonaceae collection plot, ca. 5 m alt., 23 June 2022, *P. Chalermglin 650623/1* (originally from Phu Muang temple, Lang Suan District, Chumphon Province, Peninsular Thailand, ca. 100 m alt.) (PSU).

#### Discussion.

*Mitrephoralangsuanensis* resembles *M.macclurei* Weerasooriya & R.M.K.Saunders, but differs in having leaves with or without domatia on abaxial leaf surface (absent in *M.macclurei*), and a densely hairy indument abaxially (*vs* sparsely hairy). The flowers of *Mitrephoralangsuanensis* have yellow outer petals with pink stripes (*vs* yellow petals without pink stripes in *M.macclurei*), margins that undulate with age (not undulate in *M.macclurei*), densely hairy indument abaxially (*vs* sparsely hairy), more carpels (10–12 *vs* 7–8) and longer fruiting pedicels (20–25 mm *vs* ca. 13 mm).

*Mitrephoralangsuanensis* also resembles *M.wangii* Hu from China (Weerasooriya and Saunders 2010), but differs in its leaf laminas that are densely pubescent (sparsely hairy in *M.wangii*), sometimes with domatia at axils of secondary veins abaxially (absent in *M.wangii*), outer petals with pink stripes (absent in *M.wangii*), longer fruiting pedicels (10–16 mm in *M.wangii*) and longer monocarp stipes (9–13 mm in *M.wangii*).

### ﻿ Mitrephora sirindhorniae

Taxon classificationPlantaeMagnolialesAnnonaceae

Chalermglin, Leerat. & R.M.K.Saunders sp. nov.

03D709FF-E423-5561-901D-46E5D4BF8A3D

urn:lsid:ipni.org:names:77311808-1

Figs 4 , 5

#### Diagnosis.

*Mitrephorasirindhorniae* resembles *M.tomentosa* Hook.f. & Thomson, but is distinguished by its leaves that are sparsely hairy abaxially, larger sepals (8–10 by 10–12 mm), larger outer petals (40–60 by 22–35 mm), larger inner petals (14–16 by 14.5–15 mm), longer flowering pedicels (25–27 mm), shorter monocarp stipes (2.5–8 mm) and monocarps with a longitudinal ridge.

**Figure 4.**
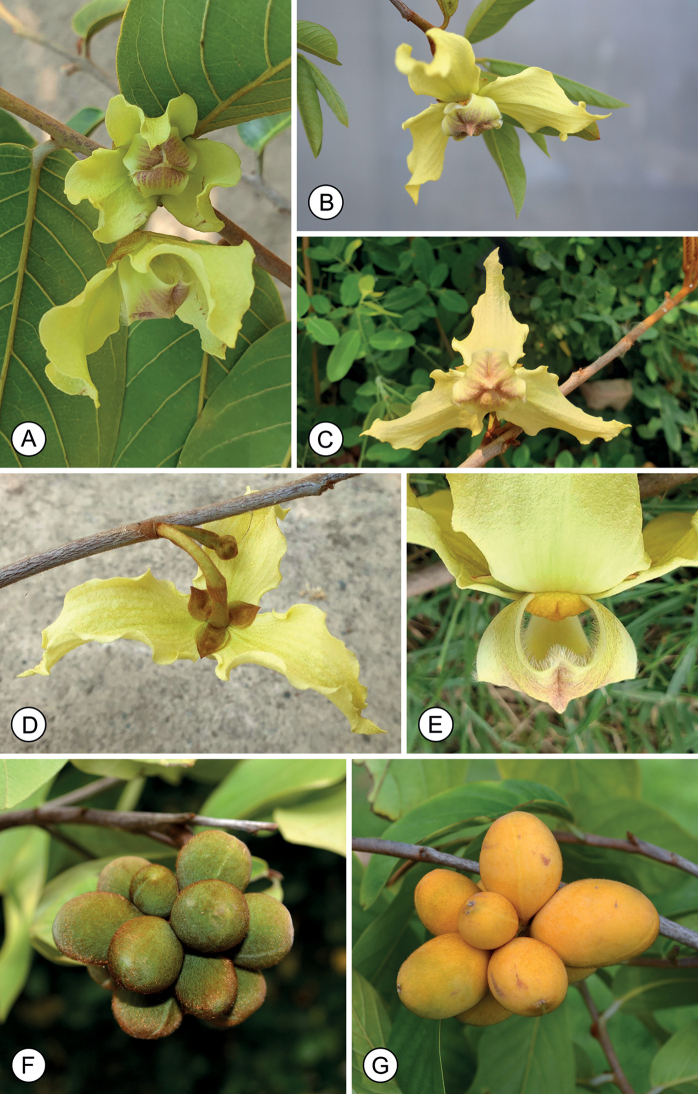
*Mitrephorasirindhorniae* sp. nov. **A** flowering branch **B, C** flowers **D** flower, showing calyx **E** flower, showing indument on adaxial surface of inner petals **F** fruit (slightly immature) **G** fruit (mature). Photos by P. Chalermglin.

#### Types.

**Thailand**: TISTR Annonaceae collection plot, Khlong Luang district, Pathum Thani Province, Central Thailand, ca. 5 m alt., 30 April 2021, *P. Chalermglin 640430* (originally from small sandstone hill in Bueng Kan Province, North-Eastern Thailand, ca. 200 m alt.) (holotype PSU; isotypes BKF, KKU, QBG).

**Figure 5.**
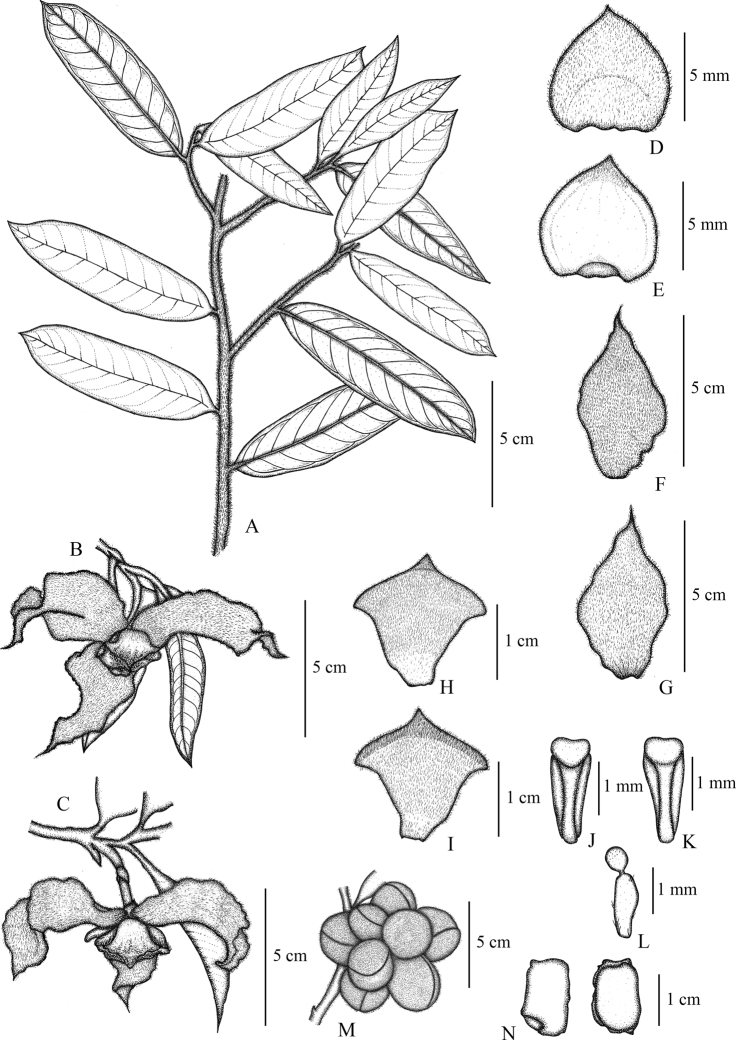
*Mitrephorasirindhorniae* sp. nov. **A** vegetative branch **B, C** flowers **D, E** sepals (ab- and adaxial) **F, G** outer petals (ab- and adaxial) **H, I** inner petals (ab- and adaxial) **J, K** stamens (ab- and adaxial) **L** carpel **M** fruit, composed of separate monocarp **N** seeds. Drawn by A. Somphrom, **A–L** from *P. Chalermglin 640430* (PSU), **M–N** from *P. Chalermglin 630723* (PSU).

#### Description

**(from cultivated material).** Small trees, to 4 m (in cultivation). Young branches densely pubescent. Leaf laminas subcoriaceous, (oblong-)lanceolate, 7–15 by 1.5–6 cm, base slightly oblique, broadly cuneate to slightly rounded, apex acute to rarely acuminate, glossy, glabrous adaxially, sparsely pubescent (denser on midrib) abaxially, secondary veins 8–12 pairs per leaf, without domatia; petioles 4–7 mm long, densely pubescent. Inflorescence rachides simple, with rachis internodes remaining short, 3–5 mm long, 2–3-flowered, densely pubescent; pedicels 25–27 mm long. Sepals free, ovate, not imbricate, 8–10 by 10–12 mm, densely pubescent abaxially, glabrous except densely pubescent at margin adaxially. Outer petals greenish-yellow, turning yellow, (broadly) oblanceolate, 40–60 by 22–35 mm, not clawed, margin undulate with age, apex acute, sparsely pubescent ab- and adaxially. Inner petals pale yellow with purple stripes apically, 14–16 by 14.5–15 mm, claw slightly incurved, densely pubescent ab- and adaxially. Stamens 1–1.5 mm long, connective truncate, glabrous. Carpels 15–20 per flower, 2–2.5 mm long; ovary oblong 1.2–1.5 mm long, sparsely pubescent, stigma club-shaped, 0.5–0.8 mm long, hairy; ovules 8–10 per carpel. Fruits with 7–14 monocarps, borne on a pedicel 20–30 mm long, 3–5 mm wide, densely pubescent. Monocarps yellow when ripe, (ellipsoid-)obovoid to ovoid, 15–30 by 10–25 mm, smooth, with longitudinal ridge, densely pubescent; stipes 2.5–8 mm long, densely pubescent. Seeds 1–10 per monocarp, semi-lenticular (lowermost and uppermost within monocarp) or discoid (others), 9–16 by 8–9.5 mm, surface glabrous, pitted.

#### Phenology

**(in cultivation).** Flowering in April and fruiting in July.

#### Distribution and habitat.

Endemic to Bueng Kan Province, North-Eastern Thailand (Fig. 3). Growing on sandstone hill in dry dipterocarp forest; ca. 200 m alt.

#### Etymology.

Named after Her Royal Highness Maha Chakri Sirindhorn, in honour of her project on plant germplasm conservation in Thailand.

#### Local name.

Maha phrom sirinthon (มหาพรหมสิรินธร) (general).

#### Additional specimens examined

**(paratypes). Thailand**: Pathum Thani Province, Khlong Luang District, TISTR Annonaceae collection plot, ca. 5 m alt., 23 July 2020, *P. Chalermglin 630723* (originally from small sandstone hill in Bueng Kan Province, North-Eastern Thailand, ca. 200 m alt.) (PSU).

#### Discussion.

*Mitrephorasirindhorniae* is characterised by its sparsely hairy abaxial leaf surface, flowers with long outer petals (40–60 mm) that are broadly oblanceolate, and by its densely pubescent fruits with a longitudinal ridge. The species is morphologically most similar to *M.tomentosa* Hook.f. & Thomson, from which it differs as its leaves are sparsely hairy abaxially (*vs* densely hairy), with flowers with larger sepals (8–10 by 10–12 mm *vs* 5–9 by 5–9 mm), outer petals (40–60 by 22–35 mm *vs* 16–19[–34] by 7.5–18 mm), inner petals (14–16 by 14.5–15 mm *vs* 8.5–16.5 by 7–12.5 mm), and longer flowering pedicels (25–27 mm *vs* 11–23 mm). The monocarp stipes are shorter (2.5–8 mm *vs* 16.5–29[–39] mm), and the monocarps have a longitudinal ridge.

### ﻿ Mitrephora sukhothaiensis

Taxon classificationPlantaeMagnolialesAnnonaceae

Leerat., Chalermglin & R.M.K.Saunders sp. nov.

7649BDD1-EE19-5E59-8B1E-29B51FE6CACA

urn:lsid:ipni.org:names:77311809-1

Figs 6 , 7

#### Diagnosis.

*Mitrephorasukhothaiensis* is distinct from all other species in having a combination of outer petals that reflex at maturity as well as inner petals that have inwardly folded marginal protrusions at the midpoint adaxially.

**Figure 6.**
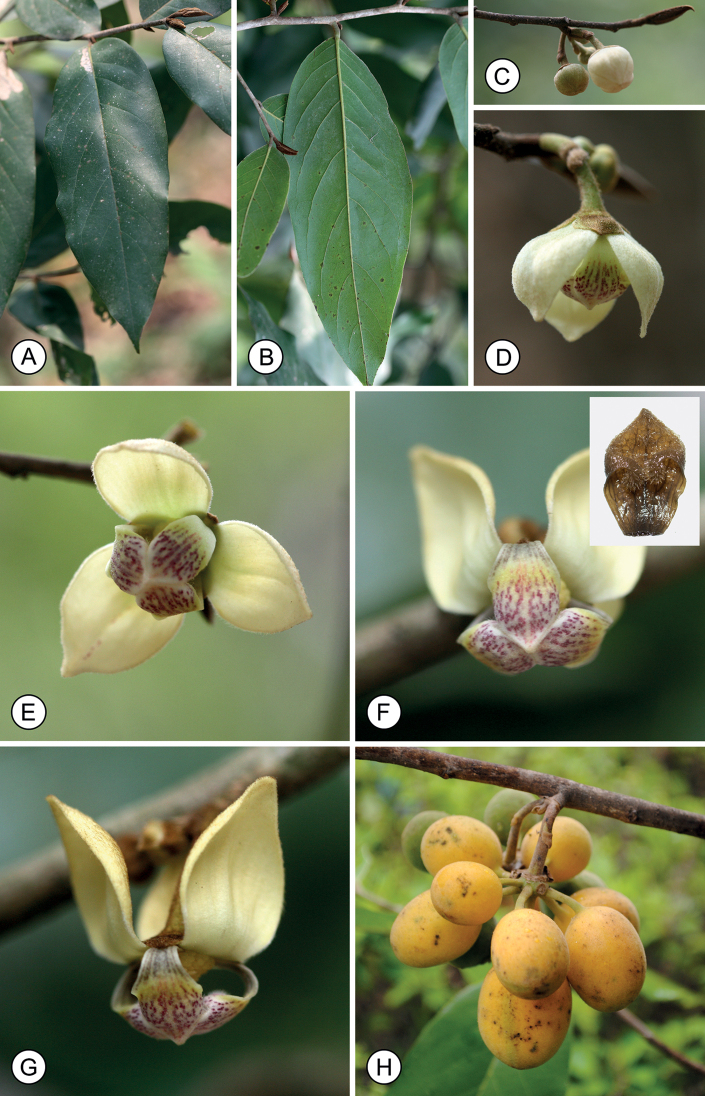
*Mitrephorasukhothaiensis* sp. nov. **A, B** leaves (ad- and abaxial) **C** flower buds **D** flower (slightly immature) **E–G** flowers, **F, G** showing the outer petals reflexed when mature, with insert in **F** showing adaxial surface of inner petal with inwardly folded marginal protrusions **H** fruit (mature). Photos by P. Chalermglin.

#### Types.

**Thailand**: Central: TISTR Annonaceae collection plot, Khlong Luang district, Pathum Thani Province, Central Thailand, ca. 5 m alt., 10 December 2021, *P. Chalermglin 641210* (originally from Si Satchanalai District, Sukhothai Province, Northern Thailand, ca. 360 m alt.) (holotype PSU; isotypes BKF, KKU).

**Figure 7.**
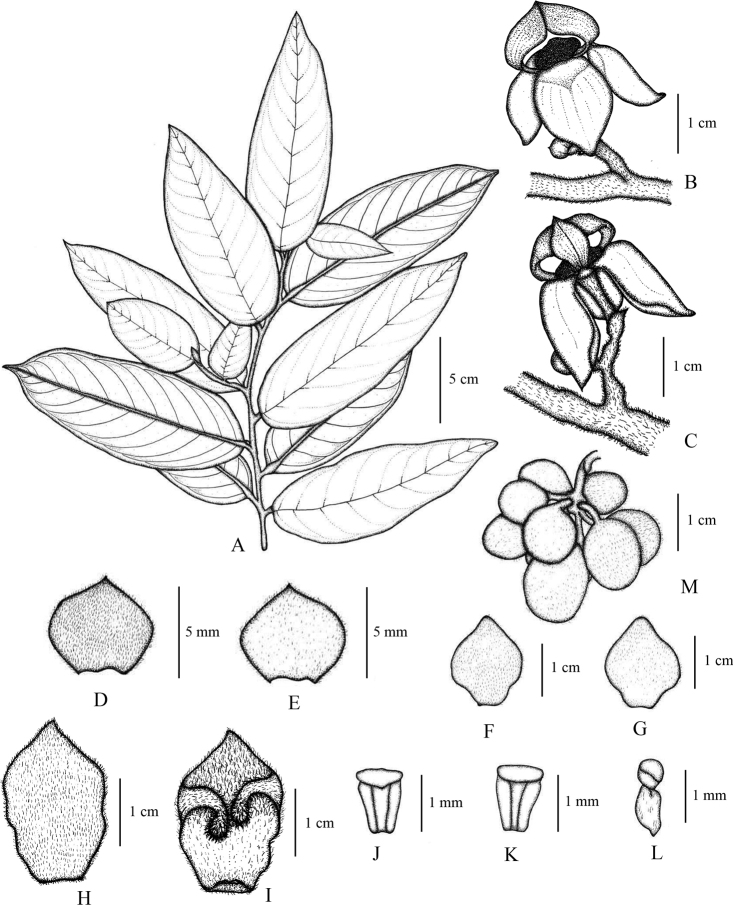
*Mitrephorasukhothaiensis* sp. nov. **A** vegetative branch **B, C** flowers **D, E** sepals (ab- and adaxial) **F, G** outer petals (ab- and adaxial) **H, I** inner petals (ab- and adaxial, showing marginal protrusions) **J, K** stamens (ab- and adaxial) **L** carpel **M** fruit, composed of separate monocarps. Drawn by A. Somphrom, **A–L** from *P. Chalermglin 641210* (PSU), **M** from *P. Chalermglin 650501* (PSU).

#### Description

**(from cultivated material).** Shrubs, to 2 m (in cultivation). Young branches densely pubescent. Leaf laminas coriaceous, (elliptic-)lanceolate or rarely ovate-lanceolate, 6–22 by 2.5–8 cm, base rounded to slightly cordate, apex acute to acuminate, glossy, glabrous (except midrib pubescent) adaxially, moderately to sparsely pubescent abaxially, secondary veins 6–14 pairs per leaf, with domatia; petioles 3–8 mm long, moderately pubescent. Inflorescence rachides simple, with rachis internodes remaining short, 1–3 mm long, 2–3-flowered, densely pubescent; pedicels 8–17 mm long. Sepals united at base, broadly ovate, not imbricate, 4–5.5 by 4–5.5 mm, densely pubescent ab- and adaxially. Outer petals creamy white to yellow, ovate, 12–17 by 9–12 mm, reflexing when mature, not clawed, margin not undulate, apex obtuse, densely pubescent ab- and adaxially. Inner petals greenish-yellow with purple spot towards apex, 10–12 by 5–6 mm, claw incurved, densely pubescent abaxially, densely hairy with long hairs towards apex adaxially, with inwardly folded marginal protrusions at the midpoint adaxially. Stamens 0.8–1.3 mm long, connective truncate, glabrous. Carpels 12–20 per flower, 1.6–2 mm long; ovary ellipsoid to oblong-ellipsoid, 1.3–1.5 mm long, moderately hairy; stigma globose, 0.3–0.5 mm long, hairy; ovules 6–10 per carpel. Fruits with up to 16 monocarps, borne on a pedicel 15–25 mm long, 4–6 mm wide, densely pubescent. Monocarps yellow when ripe, (ellipsoid-)ovoid to subglobose, 13–20 by 10–16 mm, smooth, without longitudinal ridge, densely pubescent; stipes 15–22 mm long, densely pubescent. Seeds 2–6 per monocarp, semi-lenticular (lowermost and uppermost within monocarp) or discoid (others), 5–10 by 5–6 mm, glabrous, pitted.

#### Phenology

**(in cultivation).** Flowering between December and March to June; fruiting between May and July.

#### Distribution and habitat.

Endemic to Sukhothai Province, Northern Thailand (Fig. 3). Growing in mixed deciduous forest; ca. 360 m alt.

#### Etymology.

From the name Sukhothai province.

#### Local name.

Phrom sukho (พรหมสุโข) (general).

#### Additional specimens examined

**(paratypes). Thailand**: Pathum Thani Province, Khlong Luang District, TISTR Annonaceae collection plot, ca. 5 m alt., 20 November 2020, *P. Chalermglin 631120* (originally from Si Satchanalai District, Sukhothai Province, Northern Thailand, ca. 360 m alt.) (SING); ibid. 1 May 2022, *P. Chalermglin 650501* (KKU, PSU); ibid., 23 June 2022, *P. Chalermglin 650623/2* (PSU).

#### Discussion.

*Mitrephorasukhothaiensis* is easily distinguished from all other species in two key characters: its outer petals that reflex at maturity, and its inner petals that have inwardly folded marginal protrusions at the midpoint adaxially.

*Mitrephorasukhothaiensis* also resembles *M.tomentosa* in the appearance of the fruit (monocarp shape and surface) and seeds, but differs in its shrubby habit, growing to 2 m in height (*vs* small to medium trees to ca. 20 m), sparsely to moderately hairy leaf indument abaxially (*vs* densely hairy), outer petals that reflex at maturity and without undulate margins), inner petals with inwardly folded marginal protrusions, and narrower seeds (5–6 mm *vs* ca. 8 mm).

### ﻿Key to *Mitrephora* species in Thailand

**Table:** 

1a	Outer petals 37.5–60 × 22–53 mm; inner petals 14.5–41 mm wide	**2a**
2a	Outer petals (broadly) oblanceolate, sparsely pubescent abaxially; monocarps 15–30 mm long, with 1–10 seeds	***Mitrephorasirindhorniae* Chalermglin, Leerat. & R.M.K.Saunders, sp. nov.**
2b	Outer petals ovate to broadly ovate, densely pubescent abaxially; monocarps 50–58(–68) mm long, with 13–21 seeds	**3a**
3a	Leaf laminas glossy adaxially, with 8–11 pairs of secondary veins; flower pedicels 18–27 mm long; sepals 13.5–15.5 × 14–19.5 mm; outer petals 44–53.5 × 41–53 mm; inner petals 37–43 × 36.5–41 mm	***Mitrephorasirikitiae* Weeras., Chalermglin & R.M.K.Saunders**
3b	Leaf laminas matt adaxially, with 11–13 pairs of secondary veins; flower pedicels 10–15.5 mm long; sepals 7.5–10.5 × 7.5–11 mm; outer petals 37.5–40 × 22–29.5 mm; inner petals 28–32 × 22–24.5 mm	***Mitrephorawinitii* Craib**
1b	Outer petals 4.5–34.5 × 3.5–19 mm; inner petals 3–15 mm wide	**4a**
4a	Outer petals 4.5–5 × ca 3.5 mm; inner petals 5–5.5 × 4–6 mm; monocarps 9.5–10 mm long, with solitary seed	***Mitrephorachulabhorniana* Damth., Aongyong & Chaowasku**
4b	Outer petals 9.5–34.5 × 6.5–20 mm; inner petals 6–19 × 3–12.5 mm; monocarps 11–38 mm long, with 4–10 seeds	**5a**
5a	Young branches sparsely pubescent	**6a**
6a	Flowers and fruits with a solitary carpel; monocarp sessile	***Mitrephoramonocarpa* R.M.K.Saunders & Chalermglin**
6b	Flowers and fruits with 12–16 carpels; monocarps stipitate	**7a**
7a	Flower pedicels 10–16 mm long; sepals 1.5–2.5 mm long; outer petals white, 10.5–15.5 mm wide; inner petals white with pink/purple margins, 9–14.5 × 5.5–11 mm; monocarps warty, with longitudinal ridge; stipes 6–15 mm long	***Mitrephoraalba* Ridl**
7b	Flower pedicels 4.5–9 mm long; sepals 3–4 mm long; outer petals yellow, 6.5–9.5 mm wide; inner petals yellow with pink margins, 7.5–9.5 × 4.5–6 mm; monocarps smooth, without longitudinal ridge; stipes 3–3.5 mm long	***Mitrephorakeithii* Ridl**
5b	Young branches densely pubescent	**8a**
8a	Shrubs to ca. 2 m; outer petals reflexing when mature; inner petals with inwardly folded marginal protrusions at midpoint adaxially	***Mitrephorasukhothaiensis* Leerat., Chalermglin & R.M.K.Saunders, sp. nov.**
8b	Trees to 4–30 m; outer petals not reflexing when mature; inner petals without inwardly folded marginal protrusions at midpoint adaxially	**9a**
9a	Inflorescence rachides with internodes that elongate, bearing > 3 flowers; 36–40 carpels per flower; monocarps not glaucous	***Mitrephoravulpina* C.E.C.Fisch.**
9b	Inflorescence rachides with internodes that do not elongate, bearing < 3 flowers; 7–17 carpels per flower; monocarps glaucous	**10a**
10a	Leaf laminas densely pubescent abaxially; sepals 5–9 × 5–9 mm; monocarps globose	***Mitrephoratomentosa* Hook.f. & Thomson**
10b	Leaf laminas subglabrous to sparsely pubescent abaxially; sepals 1.5–6 × 2–6.5 mm; monocarps obovoid, ellipsoid or oblong	**11a**
11a	Leaf laminas matt adaxially, inner petals cream, 6–12.5 mm long; monocarps without longitudinal ridge, densely pubescent	***Mitrephorateysmannii* Scheff.**
11b	Leaf laminas glossy adaxially; inner petals purplish, 11–19 mm long; monocarps with longitudinal ridge, sparsely pubescent	**12a**
12a	Leaf laminas densely pubescent, sometimes with domatia at axils of secondary veins abaxially; outer petals with pink stripes; fruit pedicels 20–25 mm long	***Mitrephoralangsuanensis* Leerat., Chalermglin & R.M.K.Saunders, sp. nov.**
12b	Leaf laminas sparsely pubescent, without domatia at axils of secondary veins abaxially; outer petals without pink stripes; fruit pedicels 10–16 mm long	**13a**
13a	Leaves with 7–9 pairs of secondary veins; margin of outer petals not undulate with age; petals and fruit pedicels sparsely pubescent abaxially; stipes 14–18 mm long	***Mitrephoramacclurei* Weeras. & R.M.K.Saunders**
13b	Leaves with 10–14 pairs of secondary veins; margin of outer petals ± undulate with age; petals and fruit pedicels densely hairy abaxially; stipes 9–13 mm long	***Mitrephorawangii* Hu**

## Supplementary Material

XML Treatment for Mitrephora langsuanensis

XML Treatment for Mitrephora sirindhorniae

XML Treatment for Mitrephora sukhothaiensis
